# The relation between sociodemographic factors and fatigue among urological nurses

**DOI:** 10.3389/fpubh.2025.1642002

**Published:** 2025-09-01

**Authors:** Katarzyna Jarosz, Agnieszka Młynarska

**Affiliations:** Department of Gerontology and Geriatric Nursing, School of Health Sciences, Medical University of Silesia, Katowice, Poland

**Keywords:** fatigue, nurse, urology, sociodemographic factors, MFIS

## Abstract

**Background:**

Fatigue is a multifaceted and intricate condition resulting from high demands at work and within the healthcare system, as well as from personal factors and inadequate energy recovery. It can be triggered by a variety of predictors, including lifestyle choices, environmental conditions, and both physical and mental health issues. A particularly significant factor contributing to fatigue among nurses has been the COVID-19 pandemic, which has profoundly impacted their lives, work, and perspectives on their profession.

**Purpose:**

The aim of the study was to examine the fatigue levels among nurses working in urological wards and the sociodemographic factors related to them.

**Methods:**

This is a cross-sectional study among 130 urological nurses. The questionnaire included the modified fatigue impact scale (MFIS) and metrics.

**Results:**

In our study, the mean fatigue rating was 52.68 (±16.06) points, and in the individual areas of the MFIS questionnaire, it was 24.10 (±7.34) points in physical functioning, 25.46 (±8.44) points in cognitive functions, and 5.76 (±2.13) points in psychosocial functions, suggesting that fatigue levels range between “sometimes” and “often” factors such as marital status, the number of illnesses among nurses, and the patient-to-nurse ratio are associated with the level of fatigue.

**Conclusion:**

There is a need to conduct more studies that will penetrate the fatigue experienced by nurses and the factors associated with it, especially the components related to marital status and health conditions among nurses. There is still a serious problem with nurse shortages, which cause fatigue and require more systemic solutions than just legal regulations.

## Introduction

The profession of nurse is highly exposed to physical, biological, and psychosocial risk factors, which negatively impact nurses’ health (1). Nurses’ work in hospitals is classified as medium-heavy. Nurses’ duties in surgical wards, including urological wards, primarily involve physical strength and resuscitation actions. Meanwhile, the mental load is related to the necessity of constant concentration, fast assessment, and urgent decision-making in response to sudden changes in patients’ conditions (2). These factors can lead to chronic fatigue and adversely affect their professional activity. The frequency of fatigue occurrence among nurses is 19.9%, and in most cases, it rates at approximately two-thirds of nurses (3).

Fatigue is a multidimensional and complex condition that is caused by excessive demands at work and health care system as well as individual and insufficient recovery of energy (4, 5). It encompasses any loss resulting from physical or mental effort at work and therefore includes both physiological and mental fatigue. Physiological fatigue includes muscle fatigue and nervous system fatigue, which are caused by exerting physical strength over time. Psychological fatigue includes boredom and fatigue that employees experience during tasks (6).

Researchers still look for reasons for fatigue, which could help in unambiguously diagnosing and treating it. It is estimated that among most people, this syndrome is not diagnosed (1).

Nurses within the same unit face similar work demands, which may lead to shared experiences of fatigue (7). However, researchers still do not fully understand how much of the variation in nurse fatigue and recovery can be linked to unit-level factors as opposed to individual differences. Specifically, there is a gap in our knowledge regarding whether a portion of fatigue and recovery among nurses is “shared” or common to those working in the same environment, suggesting that these issues could be addressed through unit-level or team interventions rather than solely focusing on individual variations in fatigue experiences (8).

Fatigue may be caused by various predictors, ranging from lifestyle and environmental factors to physical and mental health problems (4, 9). Person-dependent factors include home and social factors, as well as individual factors such as gender, sleep quality, mental state, marital status, household responsibilities, parenting, conflict, and social support (10). Married nurses, those who work shifts, and nurses with lower earnings tend to experience higher levels of fatigue. Nurses who report poor sleep quality also experience greater fatigue, whereas those without depressive symptoms and with more positive health perceptions report feeling less fatigued (11). Work-related fatigue is caused by the work environment, the nature of the job, poor organizational conditions, and company policies (10), overwork, shift work systems, stress, and mobbing (4, 9). Additionally, fatigue levels differ by department, with emergency room (ER) nurses being more likely to report high levels of fatigue than nurses in other nursing departments (11).

In relation to the above, three main areas of factors influencing fatigue among nurses can be distinguished. First is the characteristics of the work. Some research indicates notable differences in fatigue based on work schedules. Regarding shift work, studies have identified a link between quick returns (fewer than 11 h between shifts) and increased fatigue. Furthermore, there is evidence suggesting a strong connection between night shifts and fatigue. Psychosocial factors associated with work, such as job demands, also contribute to fatigue levels. Secondly, lifestyle and private life, sleep deprivation, body mass index (BMI), physical activity, caffeine intake, and smoking have all been shown to have significant correlations with fatigue. Thirdly, states of health such as depression, anxiety, and insomnia are related to fatigue (12). An additional and significantly important factor for the occurrence of fatigue among nurses was the COVID-19 pandemic, which made a mark on their lives, work, and perception of their profession. The pandemic was announced on March 11, 2020, by the world health organization as a global pandemic, which caused the biggest crisis all over the world. Every industry has been affected, especially healthcare and its employees (13). The main challenge in COVID-19 management was the shortage of highly qualified health workers, especially nurses, what means a significant increase in workload on nurses and giving up their private plans and hobbies. Additionally, the shortage of personal protective equipment, lack of effective management of COVID-19 in facilities, social media pressure, and poor perceived support negatively impacted healthcare providers’ efficiency and caused high levels of stress and worry (14). Health care providers spend many hours putting on and taking off personal protective equipment, which deepens the exhaustion (15). Changes in the way health services are provided, working hours, the risk of infection, and challenging workplace conditions cause anxiety, depression, fatigue, and social isolation. In the case of clinical nurses, a lack of breaks during shifts increased the frequency of fatigue and exhaustion and contributed to mental fatigue (4, 16). Accompanying the pandemic, it is not uncommon to face the deaths of patients, which can add to the burden and lead to increased levels of fatigue (17). The frequency of fatigue occurrence among health workers not on the front line was 56.7% (18). Earlier research showed that health workers not on the front line were more susceptible to fatigue than those on the front line. Da Silva et al. presented that the general rate of fatigue among nurses was 52% (19). Furthermore, a few studies showed a higher frequency of fatigue, fluctuating between 83.7 and 91.9% (20–22).

Kahriman et al. showed that 83% of medical errors positively correlate with nurses’ fatigue (23). Medically, fatigue is described as a condition characterized by a lower ability to work and lower efficiency, occurring after a period of mental or physical activity. Fatigue related to nurses’ work is considered a danger to their health, and it is also negatively correlated with patient safety and nursing care quality (4).

Due to the complexity of fatigue among nurses and the unquestionable impact of work and COVID-19 related factors, and the risk for patients, we made the decision to examine the fatigue levels among nurses in urology departments and to check whether sociodemographic predictors are related to them.

## Materials and methods

### Design of the study

This study is a cross-sectional study. The studied group consists of 130 urological nurses from Poland. According to the Central Statistical Office, in 2020, Poland had 160 urology wards with 3,147 beds, treating 188,831 patients. In total, there were 7,190 hospital wards in Poland, which contained 167,567 beds and treated 6,293,576 patients. The presence and capacity of urological departments are relatively limited compared to the overall number of hospital wards, leading to a narrower scope within urology. This translates to having fewer specialists, both doctors and nurses, in this specialized field compared to other areas of medicine. The study group, comprised of 130 nurses, is representative of this specialty. Additionally, in Poland, there has been a persistent decline in the number of actively practicing nurses, while the average age of nurses continues to rise. Many individuals who graduate from nursing programs do not pursue careers in the field or choose to work abroad (24). The factor that strongly influenced the group size was the reluctance of nurses to participate in these kinds of activities after the COVID-19 pandemic. The authors decided not to increase the sample size to prevent possible duplicate responses.

Questionnaires were distributed for 3 months, from March to May 2021, during the COVID-19 pandemic. Surveys were distributed in paper form at hospitals and online through Google Forms on nursing social media platforms. Participation in the study was voluntary and anonymous, and the nurses were informed before completing the form. Participation in the study was free of charge. Participants selection method was convenience sampling. The main sample biases were exactly convenience sampling bias and self-selection bias, because of the challenges in accessing medical staff and their reduced inclination to participate in research activities during the COVID-19 pandemic. The selection of the sample in this way had a high chance of success. Only nurses who expressed informed consent participated in the study or were consciously willing to take part in the online study. The inclusion criteria were the profession of a nurse, informed consent to participate, seniority of over 6 months as a nurse, and employment in the urology department. The exclusion criteria included incomplete fulfillment of the questionnaire, being employed in a department other than urology, and having less than 6 months of seniority. The Bioethics Committee of the Medical University of Silesia in Katowice (Ethical number: PCN/CBN/0052/KB/32/22) provided approval for the research study, and the study protocol was developed following the Declaration of Helsinki.

The data from the collected questionnaires were organized into Excel spreadsheets, and statistical analysis was performed using the Statistica 13.3 program. The level of statistical significance was set at *α* = 0.05. Regression analysis was used to calculate the association between sociodemographic factors and the level of fatigue.

### Research tool

The Modified Fatigue Impact Scale (MFIS) is derived from the 40-item Fatigue Impact Scale (FIS), which was originally designed to evaluate fatigue (in the last 4 weeks) in individuals with chronic illnesses, particularly multiple sclerosis (25). The scale was created by Fisk et al. in 1994. The Cronbach’s alpha for the original version was 0.81. The official Polish version was created by Gruszczak et al., who translated the scale and adapted it to Polish conditions in 2009. Polish version is characterized by high reliability, the Cronbach’s alpha score for the physical-MFIS subscale was determined to be 0.87, while the score for the cognitive - MFIS subscale was 0.85, and the score for the psychosocial - MFIS subscale was 0.69 (26). Studies conducted by other authors among nurses (27–29), nursing students (30, 31), and adults (32) have shown that this is a suitable tool for examining fatigue in groups beyond just those with multiple sclerosis. Consequently, the authors decided to use this scale.

The MFIS contains 21 statements that address three aspects of a person’s life: physical (being clumsy and awkward, rushing myself through physical activities, feeling less motivated to engage in physically demanding tasks, having trouble sustaining physical activities for longer periods, experiencing weaker muscles, feeling physical discomfort, struggling to complete tasks that require physical effort, reducing participation in physical activities, needing to rest more often or for longer than usual) cognitive (being less attentive, having difficulty concentrating for long periods, not being able to think clearly, forgetting things, struggling to make decisions, feeling less motivated to engage in tasks that require thinking, having trouble finishing sentences that involve thought, experiencing difficulty organizing your thoughts while working on tasks at home or at work, thinking more slowly than usual, and having trouble concentrating) and psychosocial (less motivation to participation in social life, limited ability to do activities outside the home). Each statement is scored from 0 to 4, contributing to a maximum possible score of 84. The breakdown of statements is as follows: 9 for physical, 10 for cognitive, and 2 for psychosocial. Respondents evaluate each of the 21 statements based on a scale where 0 means “never,” 1 means “rarely,” 2 means “sometimes,” 3 means “often,” and 4 means “almost always.” The overall score is calculated by summing the points, with higher scores indicating greater fatigue and its impact on quality of life. Currently, there are no established benchmarks for interpreting MFIS scores regarding levels of fatigue (26, 33). In this study, the MFIS scale demonstrated high reliability: physical functioning - Cronbach’s alpha 0.920, cognitive functioning - Cronbach’s alpha 0.952, psychosocial functioning - Cronbach’s alpha 0.875, and total score - Cronbach’s alpha 0.963.

### Characteristics of the studied group

Most participants in the survey were women, making up 98.5% of the respondents. The average age was 37.78 years (±11.86 years). They reported an average of 13.31 years (±12.63 years) of work experience and an average of 7.71 years (±10.26 years) of internship in the urology department. A majority, 57.7%, held a bachelor’s degree, and nearly half (46%) had no further postgraduate training. Additionally, a significant portion of the respondents reported having one job (61%) and working under a 12-h shift system (83%). For further details, please refer to Table 1.

**Table 1:** Sociodemographic characteristics of the studied group.

Characteristics		*N*	%
Gender	Female	128	98.5
	Male	2	1.5
Residential address	City	100	76.9
	Village	30	23.1
Relationship status	Married	75	57.7
	Single	45	34.6
	Divorcee	9	6.9
	Widow	1	0.8
Education	Doctorate, PhD	1	0.8
	Master’s degree	39	30
	Bachelor’s degree	75	57.7
	Secondary education	15	11.5
Pursuing further education post-graduation	Surgical Nursing Qualification Course	20	15
	Surgical Nursing Specialization	18	14
	Specialized course	44	34
	Postgraduate education	11	8
	Non-applicable	60	46
	More than 1	16	12
Operating mode	12-h shift work	108	83
	8-h shift work	7	5
	one-shift work	15	12
Count of positions held	one	79	61
	more than one	51	39

## Results

The average fatigue rating, as measured by the MFIS scale, is 52.68 points (±16.06 points) out of a possible 84 points, translating to approximately 2.51 points per question. This indicates that respondents experience fatigue between the levels of “sometimes” and “often.” In the physical domain, the average score is 24.10 points (±7.34 points) out of 36 points, equating to about 2.68 points per question. For cognitive fatigue, the average is 25.46 points (±8.44 points) out of 40 points, resulting in approximately 2.55 points per question. Finally, the average score in the psychosocial domain is 5.76 points (±2.13 points) out of 8, which corresponds to about 2.88 points per question. These three subscales consistently reflect fatigue levels that range from “sometimes” to “often.” Further details are provided in Table 2.

**Table 2:** Descriptive statistics.

MFIS	*M*	Mdn	SD	Sk.	Kurt.	Min.	Max.	D	*p*
Physical functioning	24.10	24.00	7.34	0.14	−0.18	0.00	36.00	0.07	0.200
Cognitive functions	25.46	25.00	8.44	0.35	−0.33	0.00	40.00	0.07	0.200
Psychosocial functions	5.76	6.00	2.13	0.16	−0.48	0.00	8.00	0.15	<0.001
Total scores on the MFIS	52.68	53.00	16.06	0.25	−0.17	0.00	84.00	0.07	0.200

M, average; Mdn, median; SD, standard deviation; Sk., skewness; Kurt., kurtosis; Min. and Max., the smallest and largest value of the distribution; D, statistics of the Kolmogorov-Smirnov test; p, significance; MFIS, Modified Fatigue Impact Scale.

Correlation analysis using the Spearman test showed that predictors strongly correlated with other independent variables, which must be excluded: age, seniority in the nursing profession, and seniority in the urology department. Contentment regarding financial situations correlated with place of residence and the number of nurses’ diseases, which turned out to be significant in regression analysis, leading to the exclusion of financial contentment. The number of workplaces correlated significantly with the number of patients per nurse, which was included due to its significance in regression analysis (Table 3).

**Table 3:** Analysis of correlations among predictors.

Sociodemographic factors	Age	Marital status	Place of residence	Education	Overall seniority	Seniority in urology department	Number of workplaces	Work system	Number of nurse’s diseases	Postgraduate education	Number of patients per one nurse
Marital status	0.630***	–									
Place of residence	0.101	−0.085	–								
Education	0.061	0.044	0.026	–							
Overall seniority	0.900***	0.498***	0.139	0.140	–						
Seniority in urology department	0.647***	0.285**	0.158	0.159	0.725***	–					
Number of workplaces	0.032	0.109	0.012	−0.104	0.015	−0.015					
Work system	0.213*	0.085	0.162	0.020	0.235**	0.208*	−0.120				
Number of nurse’s diseases	0.029	−0.067	0.029	0.005	0.066	0.032	0.114	−0.135			
Postgradute education	0.281**	0.093	0.139	−0.225*	0.316***	0.290 ***	0.157	0.275**	0.003		
Number of patients per one nurse	0.124	0.050	0.121	−0.112	0.083	0.061	0.262**	−0.117	0.131	0.116	
Satisfaction with finacial situation	0.049	0.082	0.184*	−0.028	0.047	−0.027	−0.064	0.096	0.186*	−0.108	0.168

Outcomes with * *p* < 0.05; ** *p* < 0.01; *** *p* < 0.001.

Source: own study.

The F test result is statistically significant and indicates that sociodemographic factors are statistically significant related to fatigue among nurses (*F* (6.123) = 8.91; *p* < 0.001).

Analysis showed a statistically significant association between marital status and fatigue level. The *β* coefficient was −0.17; t (123) = −2.26; *p* = 0.03. This means that with a change in marital status, fatigue decreases by 0.17 points. Secondly, the analysis showed a statistically significant association between the number of nurses’ diseases and the fatigue level. The *β* coefficient was 0.43; *t* (123) = 5.64; *p* < 0.001. This means that for each additional disease, the fatigue level increases by 0.43 points. There was also a statistically significant association between the number of patients assigned to one nurse and the level of fatigue. The β coefficient was 0.19; *t* (123) = 2.40; *p* = 0.02. This means that as the number of patients per nurse increases, the fatigue level rises by 0.19 points.

The corrected R^2^ coefficient was 0.27, which means that the model explains 27% of the variance in the MFIS scale (Table 4).

**Table 4:** Regression analysis.

Model		*SS*	*df*	*MS*	*F*	*p*
MFIS	Regression	11047.09	6	1841.18	8.91	<0.001
	Rest	25416.33	123	206.64		
	Total	36463.42				
Model		*B*	*SE*	*β*	*t*	*p*
MFIS	(Constant)	17.58	4.70		3.74	<0.001
	Marital status	−4.71	2.08	−0.17	−2.26	0.03
	Place of residence	1.26	3.09	0.03	0.41	0.68
	Education	0.86	1.97	0.03	0.44	0.66
	Work system	−2.41	2.47	−0.08	−0.98	0.33
	Number of nurse’s diseases	2.97	0.53	0.43	5.64	<0.001
	Number of patients per one nurse	0.69	0.29	0.19	2.40	0.02
Model		*R^2^*		Corrected *R^2^*		The standard error of the estimate
MFIS		0.30		0.27		14.37

## Discussion

In our study, the average fatigue rating was 52.68 points, indicating that fatigue falls between the levels of ‘sometimes’ and ‘often.’ As mentioned in the introduction, the factors contributing to fatigue can be categorized into three main areas: work characteristics, nurses’ lifestyle and private life, and nurses’ health condition. In this study, the authors investigate the relationship between fatigue and sociodemographic factors, which are partly related to each of these areas. Marital status (lifestyle and private life), the number of nurses’ illnesses (health condition), and the number of patients per nurse (work characteristics) were associated with the level of fatigue. That kind of relation confirmed among Saudi nurses that work-related factors, psychosocial factors, and individual characteristics could affect or contribute to fatigue and recovery (34). The 2023 report from the Supreme Chamber of Nurses and Midwives regarding the state of nursing and midwifery indicated that Poland had 315,670 registered nurses, resulting in an average of 62 active nurses for every 10,000 residents. A significant portion of these nurses, approximately 60.4%, were employed in hospitals. Furthermore, 62.9% of nurses worked in a single position, while 27.9% held jobs at two workplaces, and 6.7% worked at three or more. Nearly one-third of registered nurses and midwives were aged 51 to 60, with another third over 60, while only one-sixth were under 40. Notably, 34% of nurses had already reached retirement age. An examination of the nursing and midwifery demographics, considering age and professional activity, reveals substantial risks to the stability of the workforce in this sector. If the number of new practitioners remains constant at 6,205, the shortage of staff is projected to increase from 2,500 in 2023 to nearly 3,900 in 2029 and 3,700 in 2030, culminating in an overall shortfall exceeding 26,000 from 2023 to 2030 (35). While these specific factors were not directly analyzed in our study, they present a profile of the Polish nursing workforce and highlight key challenges faced by this group.

The issues of staff shortages, the lack of substitutes for retiring nurses, and the aging nurse population, combined with the need for nurses to work in multiple positions, considerably weaken the profession. These challenges lead to heightened workloads, exacerbating feelings of fatigue and compounding other fatigue-related factors. Our study confirmed a correlation between increased workload, including a higher number of patients per nurse during each shift, and fatigue. Nurses cited high mental stress associated with their roles (1.9%) and challenging working conditions (2.9%) as reasons for leaving the profession, underscoring the severity of the situation. Moreover, 36.1% of nurses perceived their work as having a negative effect on their health, with 19.9% deeming the impact decidedly harmful (35). Fatigue can result from various illnesses and can also lead to health issues; long-lasting fatigue can make it difficult to determine whether specific ailments are causes or consequences of fatigue. Our study confirmed a clear link between nurses’ illnesses and fatigue levels. When asked about achieving a work-life balance, a majority of nurses expressed that it was rather impossible (28.4%), and 15.5% stated it was definitely impossible (35). This reflects how the profession significantly impinges upon nurses’ private lives, contributing to their perceived fatigue. Additionally, our own research indicated that marital status were associated with fatigue levels, highlighting the challenges of juggling personal responsibilities with professional demands and the mental and physical strain they entail.

In the research conducted by Ozvurmaz et al., nurses scored 69.18 out of 90 on the fatigue scale. In contrast, an earlier study by Celik et al., which utilized the same scale, reported fatigue scores of 70.17 out of 90 points (10). Hosseini et al. found a total fatigue score of 32.46 points, indicating above-average fatigue levels (36). Alameri et al. observed that a significant majority of nurses in Saudi Arabia experienced severe fatigue (90.2%), while this figure was 100% in China and Taiwan. In contrast, some countries exhibited lower frequencies of severe fatigue, with Norway reporting 35.4% (37). Sikaras et al. corroborated findings from studies in China, Spain, Italy, and the USA, which indicated that nurses experienced high levels of fatigue during the COVID-19 pandemic. A systematic review revealed moderate to heavy fatigue levels among nurses worldwide prior to the pandemic (38). Research by Martin et al. indicated that just over half of the respondents experienced emotional exhaustion, with 50.8% reporting this condition, while 56.4% reported physical exhaustion. A slightly smaller percentage of respondents noted experiencing fatigue (49.7%), burnout (45.1%), or exhaustion (29.4%) ‘several times a week’ or ‘every day’ (39). A study involving Polish nurses indicated that fatigue rates ranged from 35.3 to 50.5% (40). Our own study indicates a moderate level of fatigue, aligning with the findings of Ozvurmaz et al., Martin et al. and the systematic review prior to the pandemic, as well as with the fatigue frequency reported in studies from Norway and Poland. Other research has documented higher levels of fatigue than those observed in our study.

In the Dudek study, a high level of chronic fatigue was noted among nurses working in pediatric wards, which was comparable to that experienced by those in medical and surgical wards (1). This includes urology wards, as shown in our study; however, the nurses in our study exhibited a moderate level of fatigue.

The connection between somatic symptoms and overall human well-being has become increasingly apparent during the COVID-19 pandemic. Individuals experiencing somatic symptoms have faced a heightened psychological burden, which includes anxiety, depression, perceived stress, feelings of threat, diminished psychological flexibility, and persistent fears and ruminations related to the pandemic. The adverse effects of somatic symptoms are particularly pronounced in those with pre-existing mental and physical health issues. Approximately 30% of patients reported lingering physical symptoms after contracting SARS-CoV-2, which correlates with a higher likelihood of negative illness perceptions and somatic symptom disorder. Some researchers argue that Long COVID may be viewed as a somatic symptom disorder, as the consequences of the pandemic have created a ‘perfect storm’ for enduring somatic experiences (41). The impact of COVID-19 on public health was the same, or even greater, among nurses and those not working in healthcare. Nurses cared for the sick during this time, were significantly exposed to the disease, and even fell ill more often than others. The pandemic undoubtedly impacted their mental and physical well-being. Rainbow et al. reported a high prevalence of chronic fatigue among nurses, estimating it at 1,088 cases per 100,000. This prevalence can be attributed to their membership in a high-risk group that is frequently exposed to occupational stressors such as shift work, exposure to viruses, and accidents (42). Alameri et al. identified several factors influencing fatigue levels in nurses, including pain, mental health, work-related challenges, family responsibilities, cortisol levels, circadian rhythms, and total sleep duration (37). Additionally, our study noted that marital status, the patient-to-nurse ratio, and overall health status are significant factors affecting fatigue among nurses.

In the study conducted by Ozvurmaz et al., it was revealed that fatigue is more common among married individuals. This can be attributed to the dual workload faced by married nurses, both at work and at home. The study indicated that women experienced high fatigue scores and low energy levels. Despite the social transformations of the 21st century, women continue to bear the responsibility of managing household tasks, childcare, and education, as well as spousal duties (10). Conflicting demands from family life and professional obligations can further exacerbate fatigue (17). Alsayed et al. noted that parenting and family caregiving—such as the number of dependents and work–family conflict—along with adverse health conditions, can contribute to the onset of fatigue. A significant factor may be the demanding workload faced by nurses in public hospitals, who must provide caregiving services while also managing housework, caring for children, and supporting older adult relatives at home (like in-laws). As a result, the challenge of balancing work and family obligations may lead to increased fatigue (34). Supporting this observation, Huang et al. discovered that married nurses often struggle to reconcile the requirements of their professional roles with their personal lives. Consequently, nurses frequently lack adequate sleep or rest, even when off duty, due to their home responsibilities (43). Han et al. identified a notable difference in intershift recovery between married and single nurses, with single nurses showing better recovery outcomes (44). In contrast, Geiger-Brown et al. reported that nurses who are divorced, separated, or widowed had higher intershift fatigue (45). These findings, along with our study, highlight a correlation between marital status and fatigue. There are numerous factors related to marital status that contribute to fatigue and its intensity. However, the research by Karimi et al. found no significant difference in fatigue levels among the different marital status groups (46). Winwood et al. demonstrated that the relation between marital status and fatigue in nurses is inconsistent. Fatigue is believed to be associated with marital status through domestic responsibilities, which may affect recovery opportunities and lead to chronic fatigue. This inconsistency may be explained by the number of dependents, as well as the presence of relatives or support systems. Additionally, companionship can provide a means of sharing work-related stress, and partnered nurses might benefit from the calming effects of physical closeness and intimacy (47, 48). Therefore, the effect of marital status on nurses’ fatigue warrants comprehensive investigation, considering all additional factors that may be related to this status.

Alsayed et al. identified various work-related factors that contribute to nurse fatigue and recovery, particularly in public hospitals. These factors include staff shortages, higher workloads related to patient and task assignments, and heightened physical, mental, and emotional demands placed on nurses (34). Additionally, a study by Alahmadi and Alharbi in Saudi Arabia revealed that the growing demand for nurses, limited bed capacity, inadequate entries into the nursing profession, mandatory overtime, and reduced staffing levels might exacerbate nurses’ workloads (49). All of these factors are associated with the patient-to-nurse ratio, which influences nurses’ fatigue. As highlighted by Lasater et al., patient-to-nurse ratios have varied significantly across hospitals, ranging from 3.3 to 9.7 in adult medical-surgical units in recent years (50). Additionally, in a study conducted by Martin et al., 62% of the participants, comprising 29,472 registered nurses—indicated that their workload had increased during the COVID-19 pandemic (39). Hoogendoorn et al. also discovered a notable increase in the number of patients per nurse during the COVID-19 period compared to the non-COVID period. The proportion of unplanned surgical patients within the total intensive care unit population was higher during the COVID-19 timeframe than in the non-COVID period. Previous studies have indicated that the nursing workload associated with unplanned medical and surgical admissions is greater than that of planned surgical admissions (51). According to a report from the Supreme Chamber of Nurses and Midwives in Poland, 14.5% of nurses care for 1 to 5 patients; however, this low number is uncommon in surgical and medical wards. Nearly 14% of nurses manage 5 to 10 patients, with this percentage being lower among those working in surgical settings. Over 80% of nurses in medical and surgical wards care for more than 10 patients, including 14% who tend to care for more than 30 patients (35). Our study has indicated a correlation between the level of nurses’ fatigue and patient-to-nurse ratios, likely stemming from increasing patient admissions and nursing shortages, particularly during the COVID-19 pandemic.

Bordignon et al. demonstrated in their research that a significant proportion of professionals reported experiencing at least one medically diagnosed injury or illness, with over half of these individuals indicating they had two or more health issues. A study conducted in Australia examined long-term health conditions among nurses and midwives, revealing a considerable number of professionals who reported having at least one condition, with many also disclosing multiple conditions (52). Damiani et al. highlighted work-related endocrine, nutritional, and metabolic disorders, noting that 16.3% of nurses reported hypothyroidism, 8.1% reported hyperinsulinemia, and 4% were affected by diabetes mellitus (53). Rainbow et al. found that 55% of nurses were overweight, while 27.1% were classified as obese (42). Additionally, Aboagye et al. established that the presence of employees with certain diseases at work increases the risk of moderate to severe fatigue and a decline in efficiency over the past year (54). Fatigue was reported by 37% of respondents with localized pain and by 47–64% of those experiencing chronic widespread pain. In a Canadian study, participants who reported sleep disorders also experienced fatigue. Research by Hiestandt et al. indicated showed that that factors such as pain, sleep duration of less than 6 hours, insomnia, somnolence, anxiety, and depression (55) as well as pain, mental health, cortisol levels (37), occupational stress (56), and work musculoskeletal symptoms (36) were all correlated with fatigue. These health issues serve as examples of the numerous and complex health problems faced by nurses, who are particularly susceptible to a variety of illnesses. Our study corroborated that the number of health issues among nurses significantly associate with their levels of fatigue. It is worth mentioning, the relationship between somatic symptoms and human well-being has become increasingly evident during the COVID-19 pandemic. Individuals experiencing somatic symptoms reported heightened psychological distress, including anxiety, depression, perceived stress, a sense of threat, reduced psychological flexibility, and concerns and ruminations regarding the pandemic. The adverse effects of somatic symptoms were particularly pronounced in individuals with pre-existing mental and physical disorders. It was found that 30% of patients experienced persistent physical symptoms after contracting SARS-CoV-2, which were linked to an increased likelihood of negative illness perceptions and somatic symptom disorder. Some researchers have even posited that Long COVID could be viewed as a somatic symptom disorder, as the consequences of the pandemic have created a ‘perfect storm’ for ongoing somatic experiences (41). The effects of COVID-19 on public health were comparable to, or even more severe than those experienced by nurses and others outside the healthcare sector. Nurses, who tended to the ill during the pandemic, faced significant exposure to the virus and reported a higher incidence of illness compared to others. There is no doubt that the pandemic has significantly impacted their mental and physical health.

According to the study conducted by Poursadeqiyan et al., a significant correlation exists between occupational fatigue and the level of education (3). Their findings are supported by Ricci et al., who reported that individuals without a university degree tend to perceive fatigue more acutely (57). In Ozvurmaz et al.’s research, it was found that employees who graduated from medical vocational high schools and worked in internal medicine and surgery clinics for over 15 years, while on the day shift and maintaining a 40-h workweek, exhibited lower energy levels and higher fatigue scores (10). Additionally, the study by Karimi et al. corroborates this relationship (46). Conversely, our research found no relation between education and fatigue, which may be attributed to the limited variation in the participants’ educational backgrounds.

## Conclusion

There is a need to deepen the study of fatigue, and the factors related to it, especially marital status and the health issues faced by nurses, which include additional elements.

Marital status is a factor that is unchangeable from the employer’s side because it falls within the private domain of employees’ lives. The employer may implement solutions to support employees in taking care of children or disabled family members.

Although legal regulations regarding the employment of nurses have been created, there is still a persistent problem with too many patients assigned to one nurse, leading to fatigue and additional side effects. This situation requires systemic solutions rather than merely relying on legal regulations, as there are significant shortages of nursing staff.

The COVID-19 pandemic undoubtedly impacted the feelings of fatigue and the health of nurses.

### Practical implications

In the 21st century, nursing practice must be evidence-based. Management staff, such as the head nurse, healthcare directors, and nursing managers, should regularly check the findings or establish a special team dedicated to nurse health and their well-being. Additionally, they should regularly examine their level of fatigue and related factors in their workplaces, compare it to international findings, and take action to these findings. As long as the management-level staff do not understand that exhausted nurses with heavy workloads provide low-quality services, the problem of high fatigue levels among nurses will persist. There are ongoing financial shortages in medical facilities, but less efficient nurses, who make more mistakes and are frequently on sick leave, also contribute to significant financial losses.

Individual medical facilities could offer quiet rooms filled with relaxing armchairs where nurses can be alone and engage in activities of their choice, such as screaming, hitting the wall, crying, or meditating. The use of breaks should be strictly observed, and breaks should occur off the ward, where no one can interrupt a nurse. Due to Fischer et al. (58) helpful may be a break every 2 h of shift for 15–20 min and due to Geiger-Brown et al. (59) a planned nap for 20–30 min during shift. Unrestricted access to water should be available for everyone, as this is still not a standard in many medical facilities. Workplaces, including computers, desks, chairs, patients’ beds, and worktops, should be regulated to fit employees’ needs.

Marital status is a factor that the employer has no influence on but can support the employee. Medical facilities should consider supporting employee parents in organizing childcare through cooperation with kindergartens, community centers, and other organizations to ensure that employees are prepared to return to work and avoid leaving due to parental duties. For single individuals, particularly those who have experienced a divorce or the death of a spouse, employers could organize meetings and activities outside of working hours, which would help combat loneliness.

Nurses should be supported by governmental solutions, such as additional health holidays, faster appointments with specialists in the event of an illness, access to psychological help, and increased funding for medical staff health.

Future research should concentrate on a thorough analysis of the factors contributing to the rise in nurse fatigue, addressing both individual and organizational aspects. Investigating the expectations and needs of nurses would be beneficial for reducing fatigue and promoting their physical and mental well-being. These studies should take place in specific healthcare facilities as well as on national and international scales to enable comparative analysis and formulate targeted strategies for fatigue prevention.

### Limitations of the study

The main limitation was access to urological nurses and their willingness to participate due to the restrictions introduced in Poland related to the COVID-19 pandemic, which resulted in biased sampling and a limited group size. There was a risk of nurses from other departments completing the questionnaire, especially when the online version was made available in nursing groups. There is no point of reference due to the lack of previous studies in urology departments. A threat to the study was recall bias, but it is unavoidable because the scale assumes an assessment of fatigue over the last 4 weeks. The type of study conducted does not allow for a detailed assessment of the cause-and-effect relationship or the direction of that relationship, but it shows what relationships exist between factors and the level of fatigue at the time of the study. The results presented in the study may motivate further, more detailed research in this field.

## Data Availability

The original contributions presented in the study are included in the article/supplementary material, further inquiries can be directed to the corresponding author.
